# Metabolic and neuropathic outcomes following combined sodium-glucose cotransporter-2 inhibitor therapy and adjunctive acupuncture in obese patients with diabetic neuropathy: a case series

**DOI:** 10.3389/fpain.2026.1886313

**Published:** 2026-09-24

**Authors:** Yun Jin Kim

**Affiliations:** College of Medicine and Health Sciences, United Arab Emirates University, Al Ain, United Arab Emirates

**Keywords:** acupuncture, case series, diabetic peripheral neuropathy, SGLT2 inhibitor, type 2 diabetes mellitus

## Abstract

**Background:**

Obesity and type 2 diabetes mellitus (T2DM) are associated with chronic metabolic dysregulation and microvascular complications, including diabetic peripheral neuropathy (DPN). This case series describes metabolic and patient reported outcomes during ongoing empagliflozin therapy with adjunctive acupuncture treatment.

**Methods:**

The prospective observational case series included three adult participants with obesity, T2DM, and symptom defined DPN. All participants received empagliflozin 10 mg daily for at least 4 weeks before enrollment and continued treatment while receiving standardized acupuncture twice weekly for 10 weeks. Primary outcomes included body weight, body mass index (BMI), and waist circumference. Secondary outcomes included neuropathic pain intensity assessed using the visual analogue scale (VAS), sleep quality measured by a single-item sleep quality score, and fatigue assessed using the fatigue severity scale were recorded at baseline and week 10.

**Results:**

All participants completed the intervention without adverse events. The body weight decreased by 4–9 kg, BMI by 1.2–3.5 kg/m^2^, and waist circumference by 5–10 cm. VAS reduction range from 2 to 4 points, sleep quality and fatigue scores also improved consistently in all cases. Exploratory outcomes indicated enhanced mobility, reduced nocturnal neuropathic symptoms, and high treatment tolerability.

**Conclusion:**

Combined therapy with SGLT2 inhibitors and acupuncture was associated with improvements in metabolic, neuropathic, and functional outcomes in obese patients with T2DM, and DPN. These findings suggest that a potential multidimensional therapeutic approach, however, larger controlled studies are required to confirm efficacy.

## 1 Introduction

Obesity and type 2 diabetes mellitus (T2DM) are chronic, interrelated metabolic disorders characterized by persistent insulin resistance, dysregulated energy homeostasis, and low-grade systemic inflammation (1). They contribute to progressive metabolic deterioration involving multiple organ systems, particularly microvascular network (2).

A key complication of long-standing T2DM is diabetic peripheral neuropathy (DPN), which arises primarily from a combination of hyperglycemia-induced oxidative stress (3), advanced glycation end-product accumulation (4), endothelial dysfunction (5), and impaired microvascular perfusion of peripheral nerves (6). These pathological processes lead to structural and functional nerve damage, including axonal degeneration (7), demyelination, and reduced nerve conduction velocity (8).

Clinically, DPN is one of the most prevalent and debilitating complications of diabetes, affecting approximately one-third of patients with diabetes over their lifetime (9). It is characterized by a symmetrical distal “stocking–glove” distribution of symptoms, including burning pain, paranesthesia, numbness, and hypersensitivity. In advanced stages, patients may develop sensory loss, increasing the risk of foot ulceration, infection, and lower limb amputation (10). The neurological manifestations, DPN has profound impacts on quality of life. Chronic neuropathic pain is strongly associated with sleep disturbance, reduced mobility, impaired gait stability, increased risk of falls, and psychological comorbidities such as anxiety and depression (11, 12). These combined effects significantly reduce functional independence and overall health-related quality of life.

Despite current pharmacological options, including antidepressants, anticonvulsants, and topical agents, treatment outcomes remain suboptimal, with many patients experiencing incomplete pain relief or dose-limiting adverse effects (13, 14). There is growing interest in adjunctive and integrative therapeutic approaches that target both metabolic dysfunction and neurovascular impairment.

Sodium-glucose cotransporter-2 (SGLT2) inhibitors, including Empagliflozin, have emerged as an important class of glucose-lowering agents with broad cardiometabolic benefits beyond glycemic control (15). By inhibiting renal glucose reabsorption in the proximal tubule (16), these agents promote urinary glucose excretion (17), resulting in improved glycemic profiles, reduced insulin resistance, and a modest but consistent reduction in body weight through negative energy balance (18).

Large-scale cardiovascular outcome trials have further demonstrated that SGLT2 inhibitors reduce the risk of major adverse cardiovascular events, hospitalization for heart failure, and progression of chronic kidney disease in patients with T2DM (19, 20). These benefits are thought to arise from multiple mechanisms, including improved hemodynamic status, reduced intravascular volume, enhanced myocardial efficiency, and attenuation of systemic inflammation and oxidative stress (21).

In addition to their cardiovascular and renal protective effects, SGLT2 inhibitors have been associated with modest improvements in adiposity, particularly visceral fat reduction, which may contribute to their metabolic benefits in obese individuals (22, 23). However, these favorable systemic effects, their direct impact on microvascular complications such as DPN remains limited and incompletely characterized (24).

Current evidence suggests that while SGLT2 inhibitors may indirectly influence neuropathic progression through improved glycemic stability, weight reduction, and reduction in oxidative stress, they do not consistently provide clinically meaningful relief of neuropathic pain or sensory dysfunction (25). Patients with DPN often continue to experience persistent symptoms, including burning pain, paranesthesia, and sensory loss, even when glycemic targets are achieved (26). This therapeutic gap highlights an unmet clinical need for adjunctive interventions that specifically target neurovascular dysfunction, pain modulation pathways, and nerve regeneration processes, beyond metabolic control alone.

Acupuncture has been increasingly investigated as a complementary and adjunctive therapeutic approach in the management of chronic neuropathic pain and metabolic disorders (27–29), including obesity and type 2 diabetes mellitus. Its growing clinical interest is supported by accumulating preclinical and clinical evidence suggesting multi-system physiological effects rather than isolated symptomatic relief.

The primary proposed mechanisms involve modulation of the autonomic nervous system (ANS), where acupuncture appears to influence the balance between sympathetic and parasympathetic activity (30, 31). Through somatosensory afferent stimulation and central integration within brainstem and hypothalamic regions, acupuncture may help regulate autonomic outflow, thereby improving cardiovascular variability, reducing sympathetic overactivity, and stabilizing metabolic homeostasis. This autonomic regulation is particularly relevant in diabetic neuropathy, where autonomic dysfunction often coexists with sensory nerve impairment (32).

Additionally, acupuncture has been associated with improvements in peripheral microcirculation. Experimental studies suggest that needle stimulation may induce local vasodilation via nitric oxide-mediated pathways, enhance endothelial function, and improve capillary perfusion (33). These vascular effects may contribute to better oxygen and nutrient delivery to peripheral nerves, potentially mitigating ischemic and metabolic injury that underlies diabetic neuropathy progression. Another key proposed mechanism is acupuncture that may modulate central pain processing pathways by influencing neurotransmitter systems such as endogenous opioids, serotonin, and gamma-aminobutyric acid (GABA) (34, 35). Functional neuroimaging studies have also demonstrated altered activity in brain regions involved in pain perception, including the anterior cingulate cortex, insula, and thalamus (36, 37). These changes suggest that acupuncture may contribute to adaptive neural reorganization, thereby reducing pain hypersensitivity and improving sensory processing.

Furthermore, acupuncture may exert anti-inflammatory effects by downregulating pro-inflammatory cytokines such as interleukin-6 and tumor necrosis factor-alpha, which are implicated in both metabolic dysfunction and neuropathic pain pathogenesis (38). These multimodal effects position acupuncture as an adjunctive intervention targeting both central and peripheral components of diabetic neuropathy.

This case series explores clinical outcomes following combining SGLT2 inhibitor therapy and adjunctive acupuncture in obese patients with T2DM complicated by DPN. The rationale for this integrative approach is grounded in the recognition that pharmacological management alone, while effective in improving glycemic control and reducing cardiometabolic risk, may not adequately address the multifactorial pathophysiology and persistent symptom burden associated with diabetic neuropathy.

## 2 Methods

### 2.1 Study design

This study was designed as a prospective, observational case series conducted one of outpatient integrative private clinic in Malaysia. The objective was to describe clinical outcomes following combined pharmacological and non-pharmacological intervention in obese patients with T2DM complicated by DPN.

The case series methodology was selected due to the exploration nature of the intervention, limited prior evidence on combined therapy, and the intention to generate hypothesis-forming clinical data. The study followed principles consistent with CARE (CAse REport) reporting guidelines for transparency and reproducibility in clinical case documentation (39).

### 2.2 Clinical setting

The study was conducted in an outpatient integrative private clinic. Patients received routine clinical follow-up throughout the intervention period.

### 2.3 Participants and DPN assessment

A total of three patients were included in this case series. DPN eligibility was based on the documented medical history and a typical distal, symmetrical symptom pattern, including burning pain, paresthesia, or numbness. Neuropathic pain intensity was recorded using the VAS, however no validated DPN specific diagnostic or severity instrument was used. Objective neurological findings including reflex, monofilament, vibration, proprioception, motor strength, nerve conduction, or quantitative sensory assessment, were not available in the case series dataset. Consequently, DPN severity was characterized descriptively from symptom burden and functional complaints.

Inclusion criteria:

- Age between 40 and 60 years
- Body mass index (BMI) between 32 and 38 kg/m^2^
- Diagnosis of T2DM
- Clinically diagnosis DPN based on typical distal neuropathic symptoms, such as burning pain, paranesthesia, or numbness
- Stable use SGLT2 inhibitor therapy for at least 4 weeks prior to enrolment

Exclusion criteria:

- Acute diabetic ketoacidosis or history of recurrent ketoacidosis
- Severe renal impairment (eGFR <30 mL/min/1.73 m^2^)
- Active lower limb ulceration or infection
- Concurrent initiation of other weight-loss pharmacotherapies during the study period
- Major neurological disorders unrelated to diabetic neuropathy

Baseline demographic and clinical characteristics were recorded prior to intervention initiation. Eligibility excluded major neurological disorders unrelated to diabetic neuropathy; however, the available records did not document a standardized differential diagnostic protocol, neurologist adjudication, or systematic testing for alternative causes of peripheral neuropathy. The diagnosis should therefore be interpreted as a symptom based clinical diagnosis of DPN.

### 2.4 Intervention

All participants received a combined therapeutic regimen consisting of standard pharmacological treatment with adjunctive acupuncture management.

Pharmacological Treatment: All patients continued stable therapy with Empagliflozin 10 mg orally once daily, followed by general practitioner. Medication adherence was monitored through routine clinical follow-up and patient self-reporting. No dose adjustments were made during the observational period unless clinically indicated.

Acupuncture Management: Acupuncture was delivered as an adjunctive therapeutic intervention using a standardized and reproducible protocol administered by registered T&CM practitioners. Treatment was provided twice weekly for 10 weeks, with each session lasting approximately 30 min. Sterile, single-use filiform needles (0.3 × 40 mm, Jiajian Medical Instruments Co. Ltd, Wuxi, China) were applied, and manual stimulation was performed to elicit the needle sensations, which was considered indicative of adequate somatosensory activation. The acupuncture protocol was designed to target multiple pathophysiological domains relevant to obesity, T2DM, and DPN, including metabolic regulation, neuropathic pain modulation, and neuromuscular functional balance.

A standardized setting of acupuncture points was selected based on prior experimental and clinical evidence:

- ST36: Associated with regulation of gastrointestinal function, energy metabolism, and systemic metabolic homeostasis
- SP6: Implicated in endocrine modulation and metabolic regulation
- LR3: Commonly used for pain modulation and autonomic nervous system regulation
- LI4: Associated with central analgesic mechanisms and modulation of pain perception pathways
- GB34: Related to musculoskeletal function and neuromuscular pain modulation

These combinations of these points were selected based on evidence from experimental and clinical studies suggesting potential effects on pain perception, metabolic regulation, and neurovascular function (40–42). These effects are hypothesized to be mediated, at least in part, through modulation of Autonomic Nervous System, along with downstream influences on peripheral circulation and central pain processing pathways.

### 2.5 Clinical timeline

Before enrollment, Patient 1, 2, and 3 had histories of T2DM lasting 8, 10, and 6 years and DPN symptoms lasting 2, 3, and 1 years. Each patient had received empagliflozin 10 mg daily at a stable dose for at least 4 weeks before baseline. At week 0, body weight, BMI, waist circumference, VAS pain, SQS sleep quality, and FSS fatigue were recorded. During Weeks 1–10, empagliflozin was continued and adjunctive acupuncture was administered twice weekly for approximately 30 min per session. At week 10, the same outcomes were reassessed and adverse events were reviewed. No follow-up sessions beyond week 10 were available.

### 2.6 Outcome measures

The clinical outcomes were assessed at two predefined time points: baseline (pre-intervention) and at the end of the 10-week intervention. Outcome selection was guided by the need to capture metabolic changes and patient-centered functional changes relevant to obesity, T2DM and DPN.

### 2.7 Primary outcomes

The primary outcomes focused on objective anthropometric indicators of adiposity and metabolic status, including (i) Body weight (kg); (ii) Body mass index (BMI); (iii) Waist circumference (cm). These parameters were selected as clinically relevant surrogate markers of central and overall adiposity, and as standard endpoints in metabolic intervention studies assessing changes in obesity-related risk profiles.

### 2.8 Secondary outcomes

Secondary outcomes were designed to evaluate neuropathic symptom burden and patient-reported functional status, including (i) Neuropathic pain intensity assessed using a 10-point Visual Analogue Scale (VAS; 0–10); (ii) Subjective sleep quality, recorded using a single-item sleep quality scale (SQS) assessed in patients with chronic primary insomnia (43); (iii) Subjective fatigue quality, record using the fatigue severity scale (FSS) is a nine-item unidimensional questionnaire scored on a seven point Likert scale, with high sum scores indicating greater fatigue (44). These outcomes were included to capture multidimensional changes in symptom burden and health-related quality of life, particularly in relation to DPN, where pain and functional impairment are key determinants of disease impact.

### 2.9 Exploratory clinical outcomes

In addition to predefined primary and secondary endpoints, routine follow-up records included qualitative observations concerning mobility and walking tolerance, nocturnal neuropathic symptoms, treatment tolerability, and adverse effects. These observations were not collected using standardized instruments or prespecified operational thresholds, and quantitative measures such as walking distance and nocturnal episode counts were not available. The records also did not consistently distinguish patient self-report from clinician impression. Accordingly, these observations are reported narratively as non-standardized clinical impressions and were not included in quantitative comparisons.

### 2.10 Safety monitoring

Safety monitoring was systematically monitored throughout the study period to ensure participant safety across both pharmacological and acupuncture interventions. Surveillance focused on the identification and documentation of known and potential adverse events associated with SGLT2 inhibitor therapy as well as acupuncture treatment.

In particular, clinical monitoring included assessment for (i) genitourinary infections, which are recognized adverse effects associated with SGLT2 inhibitor therapy; (ii) symptoms of volume depletion, including hypotension, dizziness, or dehydration-related manifestations; (iii) localized adverse events related to acupuncture, such as transient pain at needle insertion sites, minor bruising, or signs of local infection. All adverse events were recorded and evaluated in terms of severity and potential relationship to the interventions. No serious adverse events were reported during the observation period, and all interventions were well tolerated by participants.

### 2.11 Data collection and analysis

Data analysis used non-inferential statistical approaches appropriate for small-sample evidence studies. Baseline and week-10 values were compared descriptively for anthropometric and patient-reported outcomes; no hypothesis testing or inferential analysis was performed. Empagliflozin was an ongoing treatment, because all patients had received a stable dose for at least 4 weeks before enrollment.

Potential confounders were incompletely characterized. Standardized baseline and follow-up measure of glycemic control, including HbA1C or fasting glucose, were not available for analysis. Completed concomitant medication lists, previous treatments for neuropathic symptoms, dietary intake, physical activity, and systematic major-comorbidity assessments were also not consistently recorded. These variables were not controlled and should be considered when interpreting the observed changes.

### 2.11 Ethical consideration

This case series was conducted as an observational study using routinely collected clinical data. In accordance with institutional guidelines, ethical approval from the Institutional Review Board was not required. All procedures were performed in accordance with the principles of the Declaration of Helsinki. Written informed consent was obtained from all participants.

## 3 Case presentations

### 3.1 Case 1

A 45-year-old male with an eight-year history of type 2 diabetes mellitus presented with a two-year history of progressively worsening diabetic peripheral neuropathy. His primary complaints included burning pain in the lower extremities, particularly at night, accompanied by significant sleep disturbance. Baseline anthropometric assessment revealed a body weight of 92 kg and a body mass index of 34 kg/m^2^. The neuropathic pain intensity was rated at 7/10 and on the visual analogue scale, with sleep quality was rated at 4/10, and high fatigue levels were 34.

The patient was maintained on Empagliflozin 10 mg daily and received adjunctive acupuncture twice weekly for 10 weeks using a standardized protocol. Following the intervention, body weight decreased to 83 kg (−9 kg), with a reduction in body mass index to 30.5 kg/m^2^ and waist circumference decreasing by −10 cm. Neuropathic pain improved from 7 to 3, accompanied by substantial improvement in sleep quality from 4 to 7, and fatigue decreased from 34 to 26. The patient also reported resolution of nocturnal pain and restoration of regular sleep patterns. No adverse events were reported.

### 3.2 Case 2

A 52-year-old female with a 10-year history of type 2 diabetes mellitus presented with a three-year history of diabetic peripheral neuropathy characterized by numbness, intermittent pain, and reduced mobility. Baseline measurements showed a body weight of 98 kg and a body mass index of 36 kg/m^2^, the neuropathic pain was rated at 7/10. The patient reported impaired mobility, moderate sleep disturbance 3/10, and the fatigue rated as 42.

She continued treatment with Empagliflozin 10 mg daily and underwent adjunctive acupuncture twice weekly for 10 weeks.

At the end of the intervention, body weight decreased to 92 kg (−6 kg), with body mass index reduced to 33.8 kg/m^2^, with a reduction in waist circumference of −7 cm. The sleep quality improved from 3 to 7, and fatigue levels decreased from 42 to 33. The patient reported improved mobility and increased tolerance for daily activities. No treatment related to adverse events were reported.

### 3.3 Case 3

A 41-year-old male with a six-year history of type 2 diabetes mellitus presented with a one-year history of mild diabetic peripheral neuropathy, primarily manifesting as paresthesia and intermitted discomfort. The baseline body weight was 85 kg with a body mass index of 32 kg/m^2^, the neuropathic pain was recorded at 5/10, with relatively preserved sleep quality 5/10, and moderate fatigue rated as 47.

The patient received Empagliflozin 10 mg daily along with adjunctive acupuncture twice weekly for 10 weeks.

Following treatment, body weight decreased to 81 kg (−4 kg), and body mass index improved to 30.8 kg/m^2^, with a reduction in waist circumference of −5 cm. Neuropathic symptoms showed mild improvement from 5 to 3. The sleep quality improved modestly from 5 to 6, and fatigue scores decreased from 47 to 31. The patient reported improved general well-being and reduced frequency of preanesthetic symptoms. No adverse events were found.

### 3.4 Patient perspective

Patient perspectives were captured only through routine follow-up reports. Patient 1 reported resolution of nocturnal pain and restoration of regular sleep; Patient 2 reported improved mobility and tolerance for daily activities; and Patient 3 reported improved general well-being and fewer paresthetic symptoms. These accounts provide contextual information but were not collected using a validated patient experience measure.

## 4 Results

### 4.1 Sociodemographic and baseline clinical characteristics

All three patients completed the full 10-week intervention without attrition or protocol deviations. The cohort comprised two males and one female, with a mean age of 46.0 years (range 41–52 years). All participants had established T2DM and clinically diagnosed with DPN. Baseline anthropometric assessment confirmed class I or II obesity (BMI 32–36 kg/m^2^), with evidence of central adiposity in all cases.

Baseline neuropathic symptom profiles were heterogeneous but clinically significant, including burning pain, distal paresthesia, numbness, nocturnal exacerbation of symptoms, poor sleep, and functional limitation. Patients with longer neuropathy duration demonstrated greater symptom complexity and functional impairment at baseline. Table 1 shows the baseline sociodemographic and clinical profile.

**Table 1:** Baseline sociodemographic and clinical profile.

Patient	Age	Sex	BMI	T2DM duration (years)	DPN duration (years)	Baseline phenotype
P1	45	M	34	8	2	Pain-predominant, poor sleep
P2	52	F	36	10	3	Sensory loss, mobility limitation
P3	41	M	32	6	1	Mild sensory neuropathy

### 4.2 Primary outcomes

Post-intervention body weight, BMI, and waist circumference values were lower than baseline values for all three patients.

Body weight decreased 4–9 kg, BMI decreased by 1.2–3.5 kg/m^2^, and waist circumference decreased by 5–10 cm. Patient 1 had the largest decreases in body weight and waist circumference. Table 2 presents the patient level primary outcomes.

**Table 2:** Primary outcomes.

Patient	Weight		Δ Weight (kg)	BMI		Waist (cm)		Δ Waist
	Baseline	Post-		Baseline	Post-	Baseline	Post-	
P1	92	83	−9	34	30.5	108	98	−10
P2	98	92	−6	36	33.8	112	105	−7
P3	85	81	−4	32	30.8	104	99	−5

### 4.3 Secondary outcomes

Neuropathic Pain (VAS): VAS scores decreased from 7 to 3 in Patient 1; from 7 to 4 in Patient 2; and from 5 to 3 in Patient 3.

Sleep Quality Scale (SQS): SQS scores increased from 4 to 7 in Patient 1; from 3 to 7 in Patient 2; and from 5 to 6 in Patient 3.

Fatigue Severity Scale (FSS): FSS scores decreased from 35 to 25 in Patient 1; from 42 to 22 in Patient 2; and from 47 to 31 in Patient 3. Table 3 presents the patient level secondary outcomes.

**Table 3:** Secondary outcomes for neuropathic pain, sleep and fatigue.

Patient	VAS		SQS		FSS	
	Baseline	Post	Baseline	Post	Baseline	Post
P1	7	3	4	7	34	26
P2	7	4	3	7	42	33
P3	5	3	5	6	47	31

### 4.4 Exploratory clinical outcomes

Routine follow-up records noted perceived improvements in mobility, walking tolerance, discomfort during daily activities, and nocturnal neuropathic symptoms. These observations were not quantified, were not assessed using standardized criteria, and did not consistently distinguish patient self-report from clinician impression; they should therefore be interpreted only as descriptive clinical impression. No adverse events related to either pharmacological therapy or adjunctive acupuncture were reported during the 10-week intervention period.

## 5 Discussion

In these three patient case series, body weight, BMI, waist circumference, neuropathic pain, sleep quality, and fatigue scores changed in a direction over 10 weeks. These concurrent descriptive changes do not establish a multidimensional treatment effect or permit attribution to either component of the combined intervention. The reduction in body weight, BMI, and waist circumferences are compatible with the established metabolic effects of SGLT2 inhibitors such as empagliflozin, which promote glycosuria-induced caloric loss (45). However, the respective contributions of empagliflozin, acupuncture, natural variation, and unmeasured co-interventions cannot be separated in this uncontrolled case series.

Neuropathic pain scores decreased while sleep quality and fatigue scores changed in directions in all three patients. These patient-controlled outcomes are clinically relevant because neuropathic symptoms may persist despite adequate glycemic control (46). Potential mechanisms proposed for acupuncture-related analgesia include modulation of central and peripheral pain pathways, endogenous opioid release, neurotransmitter regulation, and changes in microvascular perfusion (47, 48). However, the present uncontrolled observations cannot determine whether these mechanisms contributed to the recorded changes.

Furthermore, these findings support the potential value of integrative therapeutic strategies in managing complex conditions such as obesity associated with diabetic neuropathy, where metabolic and neurofunctional disturbances coexist. The combined use of pharmacological and acupuncture interventions may provide complementary benefits, addressing both underlying metabolic dysfunction and symptomatic burden.

## 6 Conclusion

In these three patient case series, metabolic measures and patient-reported pain, sleep, and fatigue scores changed in directions during the 10-week combined intervention. Given the small sample, observational design, and absence of a control group, no treatment effect or causal contribution of either intervention can be inferred. These preliminary observations support feasibility and hypothesis generation for future controlled studies.

## 7 Strengths and limitations

This study has several strengths. It provides clinical observations from routine practice, and the use of a standardized acupuncture protocol supports reproducibility. However, the small sample size limits generalizability and precludes statistical inference. The absence of a control group prevents causal attribution of the observed changes and permits placebo effects, regression to the mean, and other non-specific treatment effects. The observational design also allows potential confounding, including unmeasured lifestyle changes during the study period. In addition, the exploratory observations concerning mobility, walking tolerance, nocturnal symptoms, and functional capacity were not evaluated using standardized instruments or operational definitions, quantitative distances and episode counts were unavailable, and the records did not consistently distinguish patient self-report from clinician impression. These non-standardized clinical impressions should therefore be interpreted with due caution. Finally, the relatively short follow-up does not permit assessment of the durability of the observed changes. Future studies should include larger samples, randomized controlled trials designs, standardized functional outcomes, and longer follow-up.

## Data Availability

The original contributions presented in the study are included in the article/Supplementary Material, further inquiries can be directed to the corresponding author.
